# Acculturation and Dental Visits Among Hispanic Adults

**Published:** 2009-03-15

**Authors:** Paul I. Eke, Freder Jaramillo, Gina O. Thornton-Evans, Susan O. Griffin

**Affiliations:** Centers for Disease Control and Prevention; Centers for Disease Control and Prevention, Atlanta, Georgia; Centers for Disease Control and Prevention, Atlanta, Georgia; Centers for Disease Control and Prevention, Atlanta, Georgia

## Abstract

**Introduction:**

Acculturation may strongly influence use of or access to health services among Hispanics in the United States. We assessed the relationships between acculturation and use of oral health services among Hispanic adults in the United States.

**Methods:**

Data were analyzed from Hispanic adults aged 18 years or older who participated in the 2006 Behavioral Risk Factor Surveillance System. Hispanics were defined by self-report of Spanish or Hispanic heritage. Preference to be interviewed in English or Spanish was used as a proxy for acculturation. Having had a dental visit in the previous 12 months was used as a proxy for use of oral health services.

**Results:**

English-speaking Hispanics were more likely to have had a dental visit in the previous 12 months compared with Spanish-speaking Hispanics (crude odds ratio [OR], 1.52; 95% confidence interval [CI], 1.36-1.71). After controlling for potential confounders, language was not significantly associated with having had a dental visit (OR, 1.05; 95% CI, 0.87-1.26; *P* = .61,). The most significant predictors for having had a dental visit in the previous 12 months were sex, education, income, and having health insurance.

**Conclusion:**

Acculturation assessed by language spoken was not significantly associated with having had a dental visit in the previous 12 months among adult Hispanics in the United States. The common determinants of health care use, such as sex, income, level of education, and health insurance status, were the most significant predictors of use of oral health services among adult Hispanics.

## Introduction

The 2000 Surgeon General's report *Oral Health in America* acknowledged that oral diseases are more prevalent and severe among ethnic minority populations in the United States and that the Hispanic subpopulation has the lowest rate of dental care use of any ethnic group (1). According to estimates from the US Census Bureau, Hispanics are now the nation's largest minority, and their growth rate is much higher than that of other racial/ethnic groups; in 2005 they accounted for approximately 14.4% of the US population (2). This increase in the Hispanic population suggests that factors that influence oral care among Hispanics will influence oral care in the United States as a whole (3).

Acculturation refers to how immigrants adapt to new cultures and their customs (4), and it may influence use of or access to health services among Hispanics in the United States. Acculturation may predict poorer Hispanic health and greater health service use. For example, among Mexican Americans, language and birthplace were major predictors of cardiovascular disease risk; patients who were more acculturated had higher risk (5). Acculturation among Hispanic women in New York City increased the receipt of health services (6). Another study showed that acculturated Hispanics tended to start smoking and adopt unhealthy dietary and drinking habits, which were associated with increased cancer risk (7). A recent study reported that Spanish-speaking adults in the United States are a particularly vulnerable subset of US Hispanics, and they have far worse access to health care (8).

Acculturation, dental insurance coverage, income, education, perceived oral health, and access to and use of oral health services influence dental care among Hispanics in the United States (9). For example, some studies indicate that acculturation is a predictor of better oral health, increased use of oral health services, and more positive self-rated oral health among Hispanics. Those who speak primarily English at home (a measure of acculturation) are more likely to use oral health services and establish a regular source of dental care than those who speak mainly Spanish (10,11).

In this study, we evaluated the associations of acculturation and use of oral health services among Hispanic adults aged 18 years and older in the United States by using 2006 data from the Behavioral Risk Factor Surveillance System (BRFSS). Our purpose was to determine if acculturation significantly predicted use of oral health services among Hispanics, after controlling for the effects of several common determinants of access to and use of health care.

## Methods

BRFSS is a state-based telephone survey of major health risk behaviors, use of preventive health practices, and access to health care from a representative sample of noninstitutionalized adults (aged ≥18 years) in all 50 states, the District of Columbia, Guam, Puerto Rico, and the US Virgin Islands. The survey captures an independent probability sample of households with telephones; a detailed description of the survey method and the BRFSS questionnaires, data, and reports are available at http://www.cdc.gov/brfss. In 2006, the response rate for the survey was 51.4%, and 3 oral health questions were asked of all participants. Hispanics are defined as people who reported on the survey that they were of Hispanic heritage.

We used any dental visit in the previous 12 months as a proxy for use of oral health services. This variable was measured with the question, "How long has it been since you last visited a dentist or a dental clinic for any reason?" Predefined response categories were 1 to 12 months, more than 12 months to less than 2 years, more than 2 years to less than 5 years, or more than 5 years or never. From this question, we constructed the dependent variable as having had a dental visit in the last 12 months versus not having had a dental visit in more than 12 months (including never). Respondents who reported "don't know" or had missing data were excluded. We used language preference during the BRFSS interview (English or Spanish) as a proxy for acculturation. People who answered the BRFSS question in English were classified as acculturated, and those who responded in Spanish were classified as not acculturated.

The primary objective of the analysis was to determine if acculturation was a significant predictor of a dental visit in the last 12 months among Hispanics aged 18 years and older. Statistical analysis and data management were performed by using SUDAAN version 10 (RTI International, Research Triangle Park, North Carolina) to account for complex survey design and derive accurate standard errors. Differences were considered significant at *P* < .05. We calculated percentages and 95% confidence intervals of dental visits in the last 12 months by demographic characteristics and insurance status. We assessed bivariate relationships with χ^2^ tests. We used multivariate logistic modeling to assess the association between acculturation and dental visits, adjusted for age, sex, education, income, smoking status, marital status, and insurance status. Potential confounders were determined a priori, on the basis of previous reports in the literature. We analyzed data for 21,958 observations (which represented 28,128,393 weighted counts).

## Results

Overall, an estimated 15% of the weighted population in 2006 was Hispanic. An estimated 93% and 7% of respondents answered the 2006 survey in English and Spanish, respectively. Among those identifying as Hispanics, 55% and 45% answered the survey in English and Spanish, respectively. A larger proportion of Hispanics who answered the survey in Spanish were men, in lower income groups, had less than a high school education, lacked health care coverage, were married, and had never smoked. More Hispanics who answered the survey in English reported having had a dental visit in the previous 12 months than did those who answered in Spanish (Table 1); these proportions translated to a crude odds ratio of 1.52 (95% confidence interval, 1.36-1.71). However, after controlling for sex, age, education, smoking status, income, marital status, and health care coverage in multiple logistic regression, language preference did not significantly predict having had a dental visit in the previous 12 months. Only sex, income, education, and having health insurance were significant predictors for not having a dental visit in the last 12 months among Hispanics (Table 2).

## Discussion

We found that acculturation, as measured by Spanish language use, is not a significant predictor for use of oral health services among Hispanic adults in the United States. Common determinants of use of and access to health care, specifically sex, income, education, and health insurance coverage, were the most significant predictors for having had a dental visit in the previous 12 months. Hispanic men with low income and limited education who did not have health insurance were least likely to have had a dental visit in the previous 12 months.

The results of this study are consistent with findings from the Hispanic Health and Nutrition Examination Survey (HHANES) (12,13). Education and health insurance were the most significant predictors for dental cleanings and use of dental care among Hispanics, after controlling for other common determinants of health (14,15). In addition, acculturation predicted dental care use in the previous 5 years within specific Hispanic subgroups, such as Mexican Americans, Cuban Americans, and Puerto Ricans. Some other studies have shown that among Hispanics in the United States, acculturation is associated with better oral health, increased use of oral health services, and more positive self-rated oral health (16). Hispanics who spoke primarily English at home (a measure of acculturation) were more likely to use oral health services than were those who spoke primarily Spanish (17). However, we used BRFSS data in this study, which are different from HHANES data; HHANES was conducted in the 1980s and was restricted to residents of 5 southwestern states and Miami-Dade County in Florida and Puerto Ricans living in New York City. Controlling for other determinants of health care use, the survey period and character of the population studied can influence reported associations between acculturation and use of oral health services.

In the general population, having no health insurance, particularly dental insurance, is associated with less use of oral health services and poorer oral health (18). Consistent with this fact, we found that having no health insurance plan was significantly associated with not having had a dental visit. A recent study of Hispanic adults in the United States found that Spanish-speaking adults were less likely to have health insurance and a personal physician (8). Other studies have reported that Hispanics are considerably less likely to have health insurance than are non-Hispanic white Americans (19). Among Hispanics, foreign-born Hispanics are more likely to be uninsured, from 17% of Puerto Ricans to 35% of Mexican Americans (20). Foreign-born Hispanics also report fewer health care visits and are less likely to receive preventive care (21). In this study, Spanish-speaking Hispanics were more likely to be in the low-income group and were less educated. Because insurance is typically provided by employers, less educated Hispanics are less likely to be permanently employed and thus are also less likely to have dental insurance.

We found that approximately 62% of English-speaking Hispanics, compared with approximately 51% of Spanish-speaking Hispanics, have an annual dental visit. Consequently, oral health efforts should be directed at Hispanics in low-income groups who have low levels of education and no health insurance.

The sole use of language preference for the survey may incorrectly measure acculturation. Acculturation has traditionally been measured by combining competencies in language, ethnic identification, and nativity (22,23). Others have used a 4-item index that combined language proficiency, preferences, and use (24). In addition, Spanish-language use is a proxy for lower income and lower education in the Hispanic population.

Additional limitations must be considered in this study. First, analysis of the Hispanic sample was not further stratified by ethnic subgroups, and different subgroups have unique cultural, behavioral, and social characteristics that are associated with health care use (25). Second, participants who did not have teeth were not excluded, and having teeth is strongly correlated with dental care use. Third, the BRFSS sample excludes Hispanics who do not reside in households, those who are difficult to contact because of their work schedule, and those who refuse to participate in the survey. Also, because the survey was conducted by telephone, it excludes people without residential telephone service (eg, illegal migrants, those with very low incomes, or those who use cellular telephones exclusively). Finally, the accuracy of survey participant self-report of dental visit in the last 12 months was not validated against dental records, and their response may be subject to recall bias or the tendency to give socially desirable responses during interviews.

In conclusion, public and professional resources should attempt to increase awareness of oral health among Hispanics, improve cultural competence of health care professionals, and provide affordable dental coverage to low-income and migrant workers to increase the use of oral health services among Spanish-speaking Hispanics.

## Figures and Tables

**Table 1:** Prevalence of a Dental Visit in the Previous 12 Months Among Hispanic Adults by Selected Characteristics, United States, Behavioral Risk Factor Surveillance System, 2006

**Characteristic**	% Who Reported a Visit (95% Confidence Interval)
**Language**	
English	61.6 (59.7-63.5)
Spanish	51.3 (49.2-53.4)
**Age, y**	
18-24	54.6 (50.4-58.7)
25-34	54.3 (51.5-57.1)
35-44	60.5 (57.8-63.1)
45-64	59.7 (57.1-62.1)
≥65	54.8 (50.8-58.8)
**Sex**	
Male	53.3 (51.1-55.6)
Female	60.7 (59.0-62.4)
**Annual household income**	
<$15,000	47.0 (43.8-50.4)
$15,000-$24,999	49.5 (46.6-52.4)
$25,000-$34,999	55.9 (51.5-60.2)
$35,000-$49,999	63.3 (59.3-67.2)
≥$50,000	75.4 (72.7-78.0)
**Marital status**	
Married	59.7 (57.8-61.5)
Divorced	59.0 (54.6-63.2)
Widowed	52.1 (46.7-57.5)
Separated	49.0 (42.5-55.6)
Never married	57.4 (53.7-61.0)
Unmarried couple	46.3 (41.5-51.1)
**Education**	
Less than high school graduate	44.3 (41.7-47.0)
High school graduate	57.9 (55.2-60.5)
More than high school graduate	68.2 (66.1-70.2)
**Smoking status**	
Current smoker (every day)	51.1 (46.5-55.7)
Current smoker (some days)	52.1 (46.5-57.8)
Former smoker	56.4 (53.2-59.6)
Never smoked	58.5 (56.7-60.3)
**No. of permanent teeth removed**	
0	57.4 (55.4-59.5)
1-5	58.8 (56.5-61.1)
≥6 but not all	56.8 (52.2-61.3)
All	32.9 (26.1-40.4)
Don't know/not sure	54.2 (42.2-65.7)
Refused to answer question	28.9 (9.0-62.4)
**Dental cleaning within last 12 months**	
Yes	93.5 (92.5-94.3)
No	13.4 (11.8-15.2)
**Health care coverage**	
Yes	65.8 (64.2-67.4)
No	41.0 (38.4-43.7)
**Doctor's visit for routine checkup in last 12 months**	
Yes	66.8 (65.1-68.4)
No	41.5 (39.1-44.0)

**Table 2:** Probability of Having Had a Dental Visit in the Previous 12 Months Among Hispanic Adults by Selected Characteristics, United States, Behavioral Risk Factor Surveillance System, 2006

**Characteristic**	Odds Ratio (95% Confidence Interval)	*P* Value
**Language**		
English	1.05 (0.87-1.26)	.61
Spanish	1 [Reference]	
**Age, y**		
18-24	0.97 (0.68-1.38)	.27
25-34	0.88 (0.66-1.17)	
35-44	1.10 (0.83-1.45)	
45-64	1.00 (0.77-1.30)	
≥65	1 [Reference]	
**Sex**		
Male	1 [Reference]	<.001
Female	1.33 (1.14-1.55)	
**Annual household income**		
<$15,000	0.44 (0.33-0.58)	<.001
$15,000-$24,999	0.47 (0.37-0.60)	
$25,000-$34,999	0.57 (0.43-0.75)	
$35,000-$49,999	0.64 (0.50-0.82)	
≥$50,000	1 [Reference]	
**Marital status**		
Married	1.15 (0.87-1.51)	.66
Divorced	1.10 (0.79-1.54)	
Widowed	0.88 (0.58-1.33)	
Separated	1.08 (0.70-1.67)	
Never married	1.13 (0.81-1.56)	
Unmarried couple	1 [Reference]	
**Education^a^**		
Less than high school graduate	0.64 (0.50-0.82)	.002
High school graduate	0.81 (0.65-1.03)	
Some college	0.89 (0.71-1.13)	
Graduated from college	1 [Reference]	
**Smoking status**		
Current smoker (every day)	0.84 (0.65-1.07)	.29
Current smoker (some days)	0.91 (0.67-1.24)	
Former smoker	0.85 (0.70-1.04)	
Never smoked	1 [Reference]	
**Health care coverage**		
Yes	2.10 (1.76-2.50)	<.001
No	1 [Reference]	

a We used 4 levels of education in multivariate analysis, compared with the 3 used in descriptive analysis.

## References

[1] (2000). Oral health in America: a report of the Surgeon General.

[2] US Census Bureau. Census Bureau releases population estimates by race, Hispanic origin, and age for states and counties.

[3] Wall TP, Brown LJ (2004). Dental visits among Hispanics in the United States, 1999. J Am Dent Assoc.

[4] Marin G, Marin-VanOss B, Starika MR (1991). Research with Hispanic populations. Applied social research methods series. Volume 23.

[5] Sundquist J, Winkleby MA (1999). Cardiovascular risk factors in Mexican American adults: a transcultural analysis of NHANES III, 1988-1994. Am J Public Health.

[6] O'Malley AS, Kerner J, Johnson AE, Mandelblatt J (1999). Acculturation and breast cancer screening among Hispanic women in New York City. Am J Public Health.

[7] Otero-Sabogal R, Sabogal F, Perez-Stable EJ, Hiatt RA (1995). Dietary practices, alcohol consumption, and smoking behavior: ethnic, sex, and acculturation differences. J Natl Cancer Inst Monogr.

[8] DuBard CA, Gizlice Z (2008). Language spoken and differences in health status, access to care, and receipt of preventive service among US Hispanics. Am J Public Health.

[9] (2000). The Office of Minority Health. Assuring cultural competence in health care: recommendation for national standards and an outcome-focused research agenda. Federal Register.

[10] Solis JN, Marks G, Garcia M, Shelton D (1990). Acculturation access to care, and use of preventive services by Hispanics: findings from the HHANES 1982-1984. Am J Public Health.

[11] Richardson JL, Marks G, Solis JM, Collins LM, Birba L, Hisserich JC (1987). Frequency and adequacy of breast cancer screening among elderly Hispanic women. Prev Med.

[12] Ismail AI, Szpunar SM (1990). Oral health status of Mexican Americans with low and high acculturation status: findings from southwestern HHANES, 1982-84. J Public Health Dent.

[13] Ismail AI, Burt BA, Brunelle JA, Szpunar SM (1988). Dental caries and periodontal disease among Mexican American children from 5 southwestern states, 1982-1983. Morb Mortal Wkly Rep Surveill Summ.

[14] Ismail AI, Szpunar SM (1990). The prevalence of total tooth loss, dental caries, and periodontal disease in Mexican Americans, Cuban Americans, and Puerto Ricans: findings from HHANES 1982-1984. Am J Public Health.

[15] Ismail AI, Burt BA, Brunelle JA (1987). Prevalence of tooth loss, dental caries, and periodontal disease in Mexican American adults: results from the southwestern HHANES. J Dent Res.

[16] Patrick DL, Lee RS, Nucci M, Grembowski D, Jolles CZ, Milgrom P (2006). Reducing oral health disparities: a focus on social and cultural determinants. BMC Oral Health.

[17] Graham MA, Tomar SL, Logan HL (2005). Perceived social status, language and identified dental home among Hispanics in Florida. J Am Dent Assoc.

[18] Mejia GC, Kaufman JS, Corbie-Smith G, Rozier GR, Caplan JD, Suchindran MC (2008). A conceptual framework for Hispanic oral health care. J Public Health Dent.

[19] Monheit AC, Vistnes JP (2000). Race/ethnicity and insurance health status: 1987 and 1996. Med Care Res Rev.

[20] Smelser NJ, Wilson WJ, Mitchell F, National Research Council (2002). Racial and ethnic differences in health: recent trends, current patterns, future directions. America becoming: racial trends and their consequences.

[21] Wagner TH, Guendelman S (2000). Healthcare use among Hispanics: findings from the 1994 Minority Health Survey. Am J Manag Care.

[22] Burnam MA, Hough RL, Karno M, Escobar JI, Telles CA (1987). Acculturation and lifetime prevalence of psychiatric disorders among Mexican Americans in Los Angeles. J Health Soc Behav.

[23] Vega WA, Kolody B, Aguilar-Gaxiola S, Alderete E, Catalano R, Caraveo-Anduaga J (1988). Lifetime prevalence of DSM-III-R psychiatric disorders among urban and rural Mexican Americans in California. Arch Gen Psychiatry.

[24] Marin G, Sabogal F, Marin BVO, Otero-Sabogal R, Perez-Stable E (1987). Development of a short acculturation scale for Hispanics. Hisp J Behav Sci.

[25] Stewart DC, Ortega AN, Dausey D, Rosenheck R (2002). Oral health and use of dental services among Hispanics. J Public Health Dent.

